# Mixed-methods evaluation and enhancement of Peel Public Health’s heat-related illness emergency department visit surveillance system, 2018–2024

**DOI:** 10.14745/ccdr.v52i78a05

**Published:** 2026-07-30

**Authors:** Sean Colyer, Maureen Horn, Lydia Cheng, Jessica Deming, Monali Varia

**Affiliations:** 1 Centre for Emergency Preparedness and Response, Public Health Agency of Canada, Ottawa, ON; 2 Region of Peel Public Health, Mississauga, ON

**Keywords:** heat events, heat-related illness, extreme heat, climate change, health vulnerability, public health surveillance, surveillance evaluation

## Abstract

**Background:**

Climate change contributes to increased heat events, which are associated with negative health outcomes. Peel Public Health, a health unit in Ontario, has conducted emergency department (ED) visit surveillance during heat events since 2018 using Acute Care Enhanced Surveillance data.

**Objective:**

To optimize local public health surveillance of heat-related illnesses, a mixed-methods evaluation of system usefulness and representativeness was conducted and the system was enhanced through retrospective observational analyses.

**Methods:**

The surveillance usefulness was evaluated through semi-structured interviews with ten recipients of the surveillance product and representativeness by determining the impact of data by patient address compared to hospital address over 2018–2024. Retrospective analyses of 2018–2024 data identified associations between heat events and surveillance ED indicators. Findings informed surveillance revisions.

**Results:**

Interviewees interpreted the original surveillance product as a high-level real-time snapshot of downstream health outcomes, useful for situational awareness and understanding of whether ED visits were increasing. Recommendations included adding key messages, thresholds/ranges and subgroup information. Representativeness evaluation showed that 27.2% of ED visits by Peel residents were at non-Peel hospitals and 14.0% of Peel hospital ED visits were by non-Peel residents. Daily counts of heat-related illnesses were significantly associated with heat event days (incidence rate ratio: 6.43, 95% confidence interval: 3.86–10.71).

**Conclusion:**

A revised surveillance product was implemented for 2025 including standard deviations, aberration alerts adapted from preexisting criteria, ED visits based on hospital address and stratification by three subgroups (aged older than 65 years and younger than five years, and elevated heat vulnerability) to increase usefulness for situational awareness.

## Introduction

Climate change remains an escalating challenge. In Canada, temperatures have increased at roughly double the global mean rate between 1948–2016 (1). As a largely urban population subject to the urban heat island effect, Peel region (Brampton, Mississauga and Caledon) is particularly vulnerable (2). Future heat events and, consequently, related health impacts are expected (1–3).

The association between heat events and morbidities is documented; encompassing a broad range of both primary effects and secondary exacerbations (4–7). Several subgroups are at known elevated risk for heat-related illnesses (HRIs), including low education, seniors, racialized groups, outdoor workers and those in areas with limited tree cover (3,8,9). In Peel region, 7.8% of residents were low income (2020), 15.0% had no certificate/diploma/degree (2021), 68.8% were a visible minority (2021), 137,420 worked outdoors (2016) and seniors (older than 65 years of age) are estimated to make up 21% of the population by 2041 (3,8). Public health surveillance is critical to monitoring changes in heat exposure across subgroups and heat-related health outcomes.

Peel Public Health’s HRI surveillance system monitors HRIs by use of the Acute Care Enhanced Surveillance (ACES) chief complaint syndromic emergency department (ED) visit data in all three Peel EDs (Brampton Civic Hospital, Credit Valley Hospital, Mississauga Hospital) across seven indicators (HRIs [environmental, “ENVIRO” syndrome plus chief complaint of heat stroke, heat syncope, or heat exhaustion]; sunburn; asthma; cardiovascular; mental health; respiratory, total ED visits) (10–13). Data were defined by patient residence address (Peel region). Plots of daily counts of the indicators are juxtaposed with temperature and air quality data (14,15). The plots and data (**Appendix**, **Supplemental material**, Figure S1) are distributed to internal Peel Public Health recipients during heat warnings. To optimize surveillance for local public health situational awareness, a mixed-methods evaluation of system usefulness and representativeness was conducted and the system was enhanced through retrospective observational analyses.

## Methods

### Evaluation: Usefulness

All ten surveillance product recipients, including staff who work on surveillance and in communicating notable findings to municipal partners, physicians and the general public, participated in recorded semi-structured one-on-one one-hour interviews over Microsoft Teams. This included senior leadership and analytic staff, such as an Associate Medical Officer of Health, directors and managers, specialists and epidemiologic staff working across health protection, emergency management and public health surveillance functions. The core interview questions are given in Appendix, Supplemental material, List S1. An inductive, descriptive thematic approach by a single analyst was used to analyze the interview data. No formal qualitative coding software or existing frameworks were employed.

### Evaluation: Representativeness

The public located physically within Peel region is the population at risk monitored via Peel Public Health’s HRI surveillance. The original surveillance restricted the ACES data by address of residence, regardless of the hospital address in Ontario; however, National Ambulatory Care Reporting System data showed that more than 20% of ED visits by Peel residents in 2023 were at non-Peel hospitals (*personal communication, March 2025*). This evaluation aimed to determine the impact of this practice. Two ACES datasets, one defined by Peel patient address and one defined by Peel hospital address (2018, when Peel started HRI surveillance, to 2024), were compared to determine the proportion of ED visits by Peel residents at non-Peel hospitals and the proportion of Peel hospital ED visits by non-Peel residents.

### Enhancement: Retrospective quantitative analyses

The retrospective quantitative analyses assessed associations between heat warning days (defined as days during the heat warning (16), plus two days following) and daily ED visit counts for the surveillance indicators. For HRIs, sunburn, and dehydration, Fisher’s exact tests were conducted comparing days under a heat warning (binary exposure) and days with one or more ED visits for the surveillance indicator (binary outcome). For all indicators, negative binomial regression analyses comparing days under a heat warning (binary exposure) and daily count of ED visits (continuous outcome) were conducted (without any confounder inclusions). These analyses were conducted among all ED visits at Peel hospitals from May to September (Peel’s warmest months that experience heat warnings), 2018 to 2024, among the overall population and among the subgroups: older than 65 years; younger than five years; individuals living in areas with elevated heat vulnerability (residential five-digit postal code mapped to most likely census tract within quintiles 4 or 5 of Peel Public Health’s Heat Vulnerability Index [HVI], highest risk) (17). Prior work developed this index using principal component analysis of adaptive capacity, sensitivity and exposure.

### Enhancement: Surveillance product revision

The surveillance product revision implemented lessons learned from the above analyses. Additionally, the aim was to produce systematic alerts to ACES syndrome count aberrations as opposed to visual inspection. These alerts were conducted among all ED visits at Peel hospitals. The established ACES alert metrics were applied to the surveillance indicator ED visit daily counts among the overall population as well as three subgroups (17).

Three cumulative sum alerts were used: 1) C1: daily counts are standardized relative to mean values as z scores; C1 is the sum of z for the current day and previous seven days’ counts; 2) C2: daily counts are standardized relative to mean values as z scores; C2 is the sum of z for the current day and seven days’ counts ending two days prior to the current day; and 3) C3: sum of C2 from the current day and the previous two days, but only if the previous two days did not generate alerts.

Statistical Process Control (SPC) alerts (means and standard deviations [SDs] based on the preceding 14 days): 1) extreme: daily count is greater than three SDs above the mean; 2) on edge: any two of the last three days are greater than two SDs above the mean; and 3) trend: fifth day of five consecutive days of increasing counts. All analyses were conducted in Stata 18.0.

## Results

### Evaluation: Usefulness

All ten surveillance report recipients were interviewed and responses were organized into four themes: interpretation, actions taken, data gaps and usefulness and optimization.

Respondents thought the product was a simple, helpful, real-time snapshot of ED visits that was easy to interpret. It was seen as a sentinel, providing reassurance regarding the effectiveness of mitigation measures. Many thought the absence of thresholds or ranges made it difficult to discern aberrations and make conclusions.

The surveillance product provoked very few direct actions. Heat interventions were reported to be more anticipatory and largely already in place with the declaration of the heat warning, aimed at getting people into cool spaces.

Partners generally do not use other data sources, besides media and weather alerts, informally. The main outstanding data gap pertained to HRIs not receiving hospital care. Many recipients were mindful that certain subgroups that may be at higher risk and/or more isolated may not visit the ED (e.g., community-based seniors, newcomers, outdoor workers, underhoused).

The surveillance product was reported to be useful as a high-level communication tool for situational awareness, to ensure ED visits aren’t increasing. Some recipients could not appreciate if the product was useful given its unknown consequences. Suggestions included thresholds, subgroup information, and introductory key messages.

### Evaluation: Representativeness

The representativeness retrospective analysis contrasted full-year 2018–2024 datasets. Of the 2,804,724 ED visits made by Peel residents, 762,305 (27.2%) were at a non-Peel hospital; 331,133 of 2,373,552 ED visits at Peel hospitals (14.0%) were by non-Peel residents.

### Enhancement: Retrospective quantitative analyses

There was a total of 60 ED visits for HRIs from May to September, 2018–2024, of which 10 (15.4%) were by individuals aged older than 65 years and four (6.7%) were for children aged younger than five years. Of the 60 ED visits, 41 (68.3%) were by Peel residents, of which 18 (43.9%) were mapped to Peel Public Health’s HVI quintiles 4 or 5 (highest risk) (17). There were 1,023,873 total ED visits in this time period, of which none had missing age and 710 (0.07%) had missing postal code data (only used for the elevated heat vulnerability subgroup).

Among the overall population, 26 of the 890 days that were not part of a heat warning (2.9%) had one or more HRI ED visit, compared to 31 of the 181 heat warning days (17.1%)—a significant positive association between heat warning days and HRI ED visits (*p*=0.00, **Table 1**). This was also seen among individuals aged older than 65 years (0.6% vs. 2.8%, respectively, *p*=0.02) and individuals at elevated heat vulnerability (0.6% vs. 7.2%, *p*=0.00).

**Table 1:** Cross tables and Fisher’s exact tests between presence of a heat warning^a^ and presence of one or more surveillance indicator emergency department visits, all Peel hospital emergency department visits, May–September, 2018–2024

Surveillance indicator	No heat warning, N=890 days				Heat warning^a^, N=181 days				*p*-value^b^
	No ED visit		One or more ED visits		No ED visit		One or more ED visits		
**Overall population**									
Heat-related^c^	**864**	**97.1%**	**26**	**2.9%**	**150**	**82.9%**	**31**	**17.1%**	**0**
Sunburn	**855**	**96.1%**	**35**	**3.9%**	**167**	**92.3%**	**14**	**7.7%**	**0.03**
Dehydration^d^	816	91.7%	74	8.3%	167	92.3%	14	7.7%	0.88
**Individuals aged older than 65 years**									
Heat-related^c^	**885**	**99.4%**	**5**	**0.6%**	**176**	**97.2%**	**5**	**2.8%**	**0.02**
Sunburn	890	100%	0	0.0%	180	99.4%	1	0.6%	0.17
Dehydration^d^	861	96.7%	29	3.3%	177	97.8%	4	2.2%	0.64
**Individuals aged younger than five years**									
Heat-related^c^	888	99.8%	2	0.2%	179	98.9%	2	1.1%	0.14
Sunburn	889	99.9%	1	0.1%	181	100%	0	0.0%	1.00
Dehydration^d^	715	80.3%	175	19.7%	155	85.6%	26	14.4%	0.12
**Individuals at elevated heat vulnerability^e^**									
Heat-related^c^	**885**	**99.4%**	**5**	**0.6%**	**168**	**92.8%**	**13**	**7.2%**	**0**
Sunburn	875	98.3%	15	1.7%	174	96.1%	7	3.9%	0.08
Dehydration^d^	855	96.1%	35	3.9%	176	97.2%	5	2.8%	0.67

Abbreviation: ED, emergency department

^a^ Heat warning days defined as days when the heat warning was in effect, plus the two days following each heat warning event

^b^ Fisher’s exact test used due to low counts; significant results (*p*<0.05) are bolded

^c^ Heat-related illnesses defined as the environmental (ENVIRO) syndrome plus mention in the chief complaint of heat stroke, heat syncope, or heat exhaustion

^d^ Dehydration ED visits defined by the “DEHY” Acute Care Enhanced Surveillance (ACES) syndrome (dehydration), where age is at least three years. This is in correspondence with ACES products

^e^ Individuals at elevated heat vulnerability includes Peel residents whose home residence was mapped to quintiles 4 and 5 of Peel Public Health’s Heat Vulnerability Index (i.e., highest risk)

Among the overall population, there was a more than six-fold increase in the daily rate of HRI ED visits for heat warning days relative to days not part of a heat warning (incidence rate ratio [IRR]: 6.43, 95% confidence interval [CI]: 3.86–10.71, **Table 2**). This association was also significant among individuals aged 65+ (IRR: 4.92, 95% CI: 1.42–16.98), and individuals at elevated heat vulnerability (IRR: 12.78, 95% CI: 4.56–35.86).

**Table 2:** Negative binomial regression models (incidence rate ratios) between presence of a heat warning^a^ and daily count of surveillance indicator emergency department visits, all Peel hospital emergency department visits, May–September, 2018–2024

Surveillance indicator	Overall population		Individuals aged older than 65 years		Individuals aged younger than five years		Individuals at elevated heat vulnerability^b^	
	IRR^c^	95% CI^d^	IRR^c^	95% CI^d^	IRR^c^	95% CI^d^	IRR^c^	95% CI^d^
Heat-related^e^	**6.43**	**3.86–0.71**	**4.92**	**1.42–16.98**	4.92	0.69–34.91	**12.78**	**4.56–35.86**
Sunburn	**2.13**	**1.14–3.98**	-	-	-	-	2.15	0.8–5.43
Asthma	**0.95**	**0.91–0.99**	0.95	0.90–1.003	0.68	0.59–0.78	0.95	0.90–1.006
Cardio	0.99	0.96–1.01	0.97	0.93–1.006	0.72	0.49–1.08	0.97	0.94–1.002
Mental health	0.98	0.94–1.02	0.95	0.84–1.07	0.82	0.24–2.78	0.97	0.91–1.03
Respiratory	**0.94**	**0.89–0.99**	0.95	0.87–1.04	**0.80**	**0.71–0.91**	**0.93**	**0.87–0.995**
Dehydration^f^	0.86	0.48–1.53	0.66	0.23–1.88	0.41	0.05–3.40	-	-
Headache^g^	**0.95**	**0.91–0.99**	1.00	0.92–1.09	1.01	0.58–1.76	0.98	0.92–1.04
Total ED visits	1.00	0.99–1.01	1.00	0.98–1.02	0.96	0.92–1.01	1.00	0.99–1.02

Abbreviations: CI, confidence interval; ED, emergency department; IRR, incidence rate ratio; -, not conducted due to small counts

^a^ Heat warning days defined as days when the heat warning was in effect, plus the two days following each heat warning event

^b^ Individuals at elevated heat vulnerability includes Peel residents whose home residence was mapped to quintiles 4 and 5 of Peel Public Health’s Heat Vulnerability Index (i.e., highest risk)

^c^ Incidence rate ratios from negative binomial regressions

^d^ Confidence interval; significant results are bolded

^e^ Heat-related illnesses defined as the environmental (ENVIRO) syndrome plus mention in the chief complaint of heat stroke, heat syncope, or heat exhaustion

^f^ Dehydration ED visits defined by the “DEHY” Acute Care Enhanced Surveillance (ACES) syndrome (dehydration), where age is at least three years. This is in correspondence with ACES products

^g^ Headache ED visits defined as the “HEAD” ACES syndrome (non-traumatic undifferentiated headache)

There were negative associations between heat warning days and the daily rate of ED visits in the overall population for both asthma (IRR: 0.95, 95% CI: 0.91–0.99, Table 2) and respiratory (IRR: 0.94, 95% CI: 0.89–0.99) indicators. Similar, though generally non-significant, associations were seen among individuals aged 65+ and individuals at elevated heat vulnerability. These associations were stronger and significant among individuals aged younger than five (asthma: IRR: 0.68, 95% CI: 0.59–0.78; respiratory: IRR: 0.80, 95% CI: 0.71–0.91).

### Enhancement: Surveillance product revision

Both CuSum and SPC alerts were calculated and examined, however only the latter were adopted. The CuSum alerts were triggered for some below-average counts as a mathematical artifact and deemed unhelpful. The SPC alerts were incorporated with amendments: in addition to the “extreme” alert with the criteria of the count being more than three SDs above the mean, an alert was incorporated with the criteria of more than two SDs above the mean (Appendix, Supplemental material, Figure S2). For these alerts, and the “on edge” alert, to smooth out anomalies the preceding 30 days (instead of 14 days) were used for means and SDs. Additional “trend” alerts were incorporated, reflecting three, four and five days of consecutive increases.

## Discussion

This evaluation informed revisions to Peel’s HRI surveillance by incorporating evidence-based thresholds, new indicators, expanded subgroup analyses (older adults, younger children, vulnerable residents) and improved representativeness and automation. Recipient consultation and retrospective analyses justified its continuation.

The main conclusions from the usefulness evaluation were the recommendations of thresholds/alerts, key message summaries and data about known subgroups with elevated risk. The ACES data fields are limited (demographic data limited to sex, age and five digits of postal code; no capture of unhoused) but remained the core data source due to its real-time nature. Subgroups for those aged 65 years and older and children younger than five years were incorporated.

Since ACES data include the first five digits of each patient’s postal code, the patients were mapped to their most likely census tract and linked to Peel Public Health’s established HVI quintiles, which limited the analysis to Peel residents (17). Individuals living in HVI quintiles 4 and 5 (highest risk categories) were grouped into a new “Individuals at elevated heat vulnerability” subgroup. This subgroup was inherently limited to individuals with a Peel residence. The underhoused group is excluded from this category but would now be captured more generally through the representativeness work.

The ACES ED visits to Peel hospitals were selected as they were considered more representative of people exposed to heat within Peel than data based on Peel residents seeking care at any Ontario hospital. Each approach has trade-offs: using Peel hospital visits may miss some locally-exposed residents who sought care outside the region, while using only Peel residents may include those not exposed in Peel and would exclude those without an Ontario address, such as visitors from outside the province or those who are underhoused. Focusing on Peel hospital ED visits also better reflects those who would benefit from Peel Public Health interventions. This surveillance is not intended to be a comprehensive census of all HRI cases.

The main finding of the retrospective quantitative analyses was the strong and significant positive association between heat warning days and HRI ED visits, in the overall population as well as individuals aged older than 65 years and individuals with elevated heat vulnerability. This corresponds with extant research (4–7) and justifies the continued surveillance.

The negative associations between heat warning days and the daily rate of ED visits for both asthma and respiratory surveillance indicators were reasoned to be a spurious association stemming from the analysis being limited to May to September and these indicators usually seeing their heights in the spring and fall (18). Also, individuals with these respiratory conditions may have greater health awareness during heat events.

There were few notable findings otherwise. Interviewees were not deterred by the seemingly low counts of direct HRIs or few resultant actions. They were aware that the surveillance represented a downstream outcome that was not expected to show large aberrations related to heat events; this “negative finding” was reassurance of the region’s mitigation measures.

Additional revisions to the surveillance included (Appendix, Supplemental material, Figure S2): introductory orientation page, two indicators added (dehydration and headache), expansion to May to September (versus June–September), visualized SD ranges, expanded HRI definition corresponding with ACES and increased automation.

### Limitations

This evaluation had several limitations. The interviews were limited by an informal methodology that did not use coding software or established qualitative framework. The ACES data are limited by chief complaint information and resultant surveillance indicator algorithmic allocation. The relatively small number of HRI ED visits observed during the evaluation period limits the sensitivity of this surveillance approach for detecting temporal trends. The ACES data also has very limited fields, precluding the ability to further categorize individuals at elevated HRI risk, including underhoused individuals. Lastly the SPC alerts incorporated into the surveillance had limited specificity, often alerting to non-concerning aberrations, however still underlining notable changes.

## Conclusion

Climate change is an unremitting challenge with population health implications, requiring monitoring of changes in health impacts. Evaluation of Peel Public Health’s HRI surveillance has resulted in a better-informed and more comprehensive product launched in 2025 and represents a potential example or inspiration for other jurisdictions.

## Appendix

Supplemental material is available upon request to the author: sean.colyer@phac-aspc.gc.ca

Figure S1: Example of the original heat-related illness surveillance plots and data before evaluation

List S1: Core interview questions

Figure S2: Title page and first page of the revised surveillance product (report from July 21, 2025)
